# Medical News and Items

**Published:** 1878-02

**Authors:** 


### Medical News and Items.

THE MICHIGAN STATE BOARD OF HEALTH held its regular quarterly meeting at Lansing, January 8, 1878.

Dr. Kedzie (president of the board and chairman of the committee on special sources of danger to life and health) gave a brief statement of some experiments which he had recently made in relation to the permeability of walls and clothing, and the relation of these to the healthful conditions of houses and clothing.

Leroy Parker read a report on a proposed amendment to a law requiring the transmission by the county clerks to the secretary of state, of the names and postoffice addresses of coroners as well as those of other county officers now reported. The proposed amendment will enable the state department and the secretary of the state board of health to communicate with these officers, and to learn from them the number of sudden and violent deaths, and the causes of same, with a view to remove causes when possible. He was requested to continue his investigations, and report at next meeting. He also read a report pointing out the fact that section 6852 of the compiled laws of 1871, makes it the duty of supervisors to prosecute householders and physicians for not giving notice of cases of diseases which endanger the public health. Mr. Parker and Dr. Baker were appointed a committee to draft a circular to supervisors of townships, pointing out their duties under this particular law. Mr. Parker was requested to draft an amendment to the present law which would require the health officers of cities and villages to prosecute, in the same manner as do supervisors in townships, for any neglect to give notice of contagious diseases.

The secretary stated that diphtheria had been more prevalent than usual in this and other states, and suggested that the board issue a circular on the subject. Dr. Hitchcock was requested to prepare such circular. The causes of diphtheria were thoroughly discussed, and the opinion seemed to prevail that sewer gas, dampness, and mold had much to do in causing it, although it is a contagious disease.

Dr. Kedzie made a brief report, giving an account of experiments and tests for detection of lead in tin utensils in common use, having examined quite a number of specimens. He found about three-fourths of all the specimens examined, contained lead in considerable amount. The test which Dr. Kedzie gave for this adulteration is quite simple: Place a drop of nitric acid on the tin to be tested, and evaporate to dryness; then add a drop of iodide of potassium. If lead is present, there will be a yellow coloration. If it is not present the spot will remain white. Dr. Kedzie will examine the subject further, and report at a future meeting.

WE have to congratulate ourselves upon the readiness with which our American cotemporaries emulate our example. When the CHICAGO MEDICAL JOURNAL AND EXAMINER tried the effect of a red ribbon upon its exterior, one of its Southern neighbors hastened to follow suit. And after we had donned a more sober and less pretentious garb, the *St. Louis Medical and Surgical Journal* adopted a dress so similar that it is difficult to distinguish between the two. St. Louis has long attempted to rival Chicago in various particulars; and in this matter we have to congratulate our cotemporary upon its display of good taste. We trust that before long others may resemble us in a more essential feature, by becoming the property of the entire profession in each city, and ceasing to be the organs of a college or a clique.

